# Feasibility and Acceptability of Transcranial Direct Current Stimulation in Obsessive Compulsive Disorder

**DOI:** 10.1192/j.eurpsy.2022.54

**Published:** 2022-09-01

**Authors:** N. Fineberg

**Affiliations:** University of Hertfordshire, School Of Life And Medical Sciences, Hatfield, United Kingdom

## Abstract

Background Transcranial direct current stimulation (tDCS) has shown promise as a treatment for obsessive compulsive disorder (OCD) in a small number of trials. We performed a feasibility study to inform the development of a definitive trial, focussing on acceptability, safety, feasibility of recruitment, adherence and tolerability of tDCS and the size of any treatment-effect. Methods FEATSOCS was a randomised, double-blind, sham-controlled, cross-over multicentre study. Twenty adults with OCD received three courses of tDCS targeting the two most favourable stimulation targets; supplementary motor area (SMA), orbitofrontal cortex (OFC) and sham-stimulation, randomly allocated and delivered in counterbalanced order. Each course comprised four 20 minute-stimulations, over two consecutive days, separated by a four weeks washout period. Clinical outcomes were assessed by ‘blinded’ raters before, during and four weeks after stimulation. Results: tDCS was acceptable, well tolerated and safe; adherence was good, with few dropouts, there were no serious adverse events, and adverse effects were mostly mild. Recruitment to target was feasible. Yale-Brown Obsessive-Compulsive Scale scores numerically improved from baseline to 24 hours after final stimulation (primary outcome) across all interventional groups. The greatest effect was seen in the OFC arm. Additional significant within-group improvements in secondary outcomes occurred in the OFC, and to a lesser extent in the sham arms, but not with SMA. Discussion tDCS appears a promising potential treatment for OCD. The OFC represents the optimal target. A full-scale trial to determine optimal stimulation protocols (current, frequency, duration), longer-term effectiveness and feasibility of home delivery is indicated.

**Disclosure:**

No significant relationships.

